# Indolizidines from Actinomycetes: An Overview of Producers, Biosynthesis and Bioactivities

**DOI:** 10.3390/microorganisms12071445

**Published:** 2024-07-16

**Authors:** Janina Krause

**Affiliations:** Department of Biomedical Research, Institute of Health Research and Education, School of Human Sciences, University of Osnabrueck, 49076 Osnabrueck, Germany; janina.krause@uni-osnabrueck.de

**Keywords:** indolizidine, cyclizidine, actinomycetes, biosynthesis, bioactivity

## Abstract

Indolizidines have long been recognized for their valuable bioactivities, their common feature being a bicyclic structure connected via a nitrogen atom. Traditionally, plants have been identified as the primary producers. However, recent discoveries have revealed that certain bacterial strains belonging to the genus of actinomycetes also possess the ability to synthesize various indolizidine-based compounds. Among these strains, *Streptomyces* sp. HNA39, *Saccharopolyspora* sp. RL78, and *Streptomyces* NCIB 11649 have been identified as producers of cyclizidines, characterized by their distinctive cyclopropyl moiety. Additionally, *Streptomyces griseus* OS-3601 synthesizes a unique class of indolizidine derivatives known as iminimycins, distinguished by their rare imine-cation structure. Protoplast fusion of a *Streptomyces griseus* strain with *Streptomyces tenjimariensis* resulted in a new indolizidine named indolizomycin. This review aims to provide an overview of known bacterial indolizidine producers, summarize current knowledge regarding the biosynthesis of cyclizidines and iminimycins, and assess their respective bioactivities.

## 1. Indolizidines: Structure, Sources, and Bioactivities

**Figure 1:**
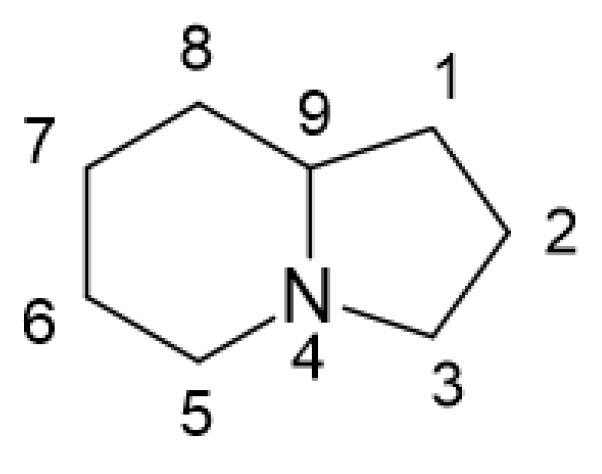
Core structure of indolizidine molecules [1].

Indolizidines represent a class of structurally related alkaloids. Their core structure consists of a bicyclic framework comprising one cyclohexane and one cyclopentane, linked together through C9 and N4 (Figure 1). Indolizidine derivatives can be extracted from various plant species, including legumes and orchids, as well as from certain animal species, such as ants, contributing to their diverse natural occurrence [1].

**Figure 2:**
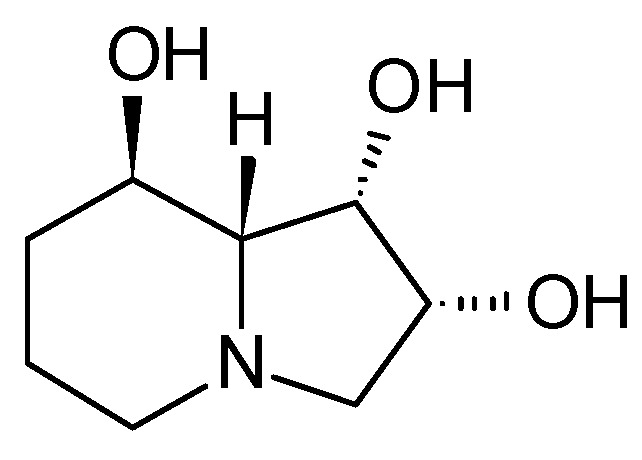
Structure of swainsonine [5].

Indolizidines display a wide range of activities, including antimycotic [2], antimalarial [3], and anti-inflammatory [4], highlighting their diverse pharmacological potential. Swainsonine from the locoweed endophyte *Alternaria oxytropi*, for example, is a well-known indolizidine notorious for its toxicity to livestock (Figure 2) [5] but also demonstrates anticarcinogenic activity. Studies have shown its ability to inhibit various glioma cancer cells [6,7] and human gastritic carcinoma cells [8], as well as to reduce the tolerance of colorectal cancer cells to 5-fluorouracil [9]. Only recently new indolizidines with an unusual, fused pyrrolidine moiety have been isolated from *Anisodus tanguticus*, a plant native to China. Some of the compounds displayed cytotoxic potential against the cancer cells lines A2780, A549, and HGC-27 [10].

## 2. Bioactive Metabolites from Microorganisms

Plants currently serve as the primary source of indolizidines [1]. However, biotechnological production by microorganisms offers several advantages over plant-based production. A broad panel of instruments exist to easily manipulate microorganisms genetically for optimized production, and their growth and production rates can be much faster compared to plants. Furthermore, the scalability of production is typically easier with microorganisms. For instance, Pyne et al. developed a yeast platform for the improved synthesis of the tetrahydroisoquinoline alkaloid (*S*)-noroclaurine. This led to manifold increases in yields and enabled the de novo synthesis of novel tetrahydroisoquinoline derivatives through genetic engineering [11]. Likewise, cultivating bacterial or fungal species capable of producing bioactive indolizidines holds great promise for unlocking prolific sources of indolizidines.

**Figure 3:**
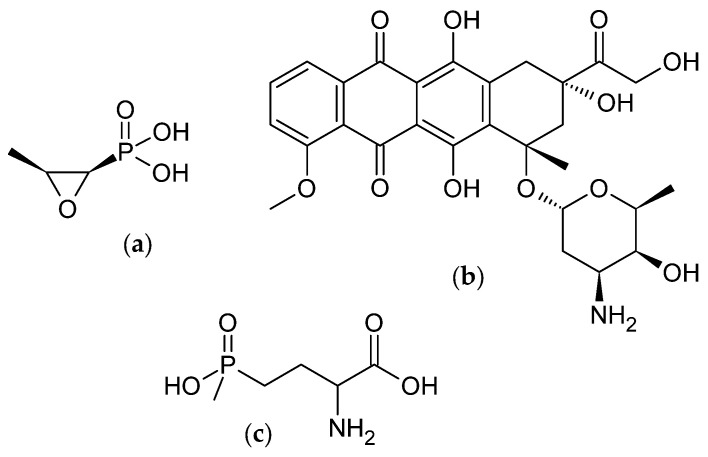
Chemical structures of the actinomycetal bioactive metabolites (**a**) fosfomycin [27], (**b**) doxorubicin [29], and (**c**) phosphinothricin [30].

As of today, five microbial species capable of producing indolizidines have been identified [12,13,14,15,16]. All five known bacterial producers of indolizidines belong to the family of actinomycetes, a diverse group of bacteria renowned for their ability to produce medically relevant natural products [17]. Actinomycetes are Gram-positive prokaryotes that typically live in aerobic environments and are apathogenic. The suffix “-myces” refers to the mycelium, which is similar in structure to that of fungi. Actinomycetes are primarily found in soil habitats but have also been discovered in marine, freshwater, desert, and volcanic environments, as well as in rhizospheres and sponges [18]. Their genome displays two unusual characteristics: on the one hand, it comprises a considerable number of base pairs (approximately 10 Mbp), and, on the other hand, it exhibits an extraordinarily high GC-content [19]. Despite recent reclassification efforts, streptomycetes remain the largest genus among actinomycetes and the most prolific secondary metabolite producers [20]. The life cycle of these organisms is complex, starting with spore-forming hyphae, which extend and branch first into the substrate and subsequently into the air [21]. Upon the depletion of nutrients, the so-formed branched hyphae can undergo septation into spores, which can initiate a new life cycle [22]. The production of secondary metabolites is typically associated with the sporulation process [23]. The most notable attribute of the actinomycete family, however, is their remarkable capacity to produce an immense array of bioactive secondary metabolites, which have been effectively employed in various fields including medicine, agriculture, and biotechnology. The first bioactive metabolite isolated from an actinomycete was streptomycin, discovered by the research group of Selman Waksman [24]. This discovery prompted a surge in research efforts aimed at identifying new bioactive agents. The “golden age of antibiotics” was largely driven by the isolation of novel actinomycete species, particularly Streptomycetes, which were evaluated for their bioactive potential through the use of simple agar diffusion assays [25,26]. Examples of medicinally and agriculturally significant secondary metabolites from actinomycetes include fosfomycin from *Streptomyces fradiae*, (Figure 3a) [27], which is considered an antibiotic of last resort by the World Health Organization [28], doxorubicin from *Streptomyces peuceticus* (Figure 3b), which is used in chemotherapy [29], and phosphinothricin produced by several *Streptomyces* species (Figure 3c), which exhibits herbicidal activity akin to glyphosate [30]. The vast potential of actinomycetes in the production of bioactive compounds with diverse applications has been extensively reviewed as, for example, in [18,31,32,33].

## 3. Known Actinomycetal Indolizidine Producers

The strains *Streptomyces* sp. HNA39 [12], Saccharopolyspora sp. RL78 [13], and *Streptomyces* sp. NCIB 11649 [16] belong to the five known bacterial producers of indolizidines. These strains produce a group of indolizidine derivatives, which are collectively known as cyclizidines, due to a cyclopropane moiety in their structure. Various cyclizidines exhibit low to moderate cytotoxicity [12,13]. Additionally, *Streptomyces griseus* OS-3601 produces iminimycins, another type of indolizidine derivative, which carry a rare imine-cation [15,34]. Lastly, one indolizidine compound was created via protoplast fusion of a *S. griseus* strain with a *Streptomyces tenjimariensis* strain resulting in a new structure named indolizomycin [14]. The following sections will present all indolizidine compounds found. Information will also be provided regarding the producers, although in the majority of cases only a few facts are known, such as the sampling source and the cultivation conditions. The focus is consistently on the indolizidines that are produced.

### 3.1. Cyclizidine M146791 from Streptomyces sp. NCIB 11649

#### 3.1.1. Structure and Isolation

**Figure 4:**
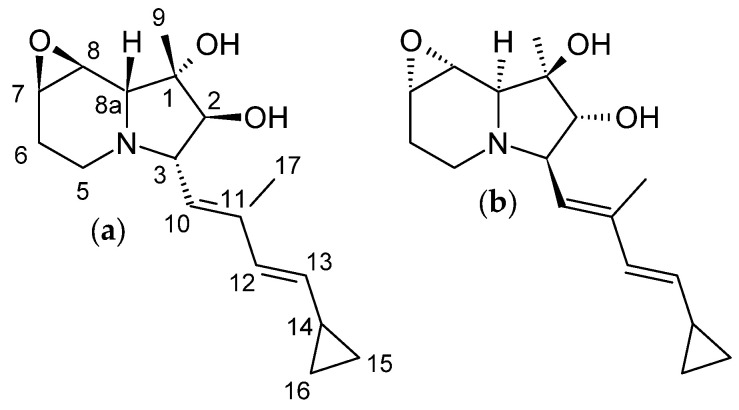
Chemical structure of the first-ever isolated cyclizidine M146791 from *Streptomyces* sp. NCIB 11649 as (**a**) originally proposed in 1987 [16] and revised in 2010 (**b**) [36].

The first cyclizidine, named M146791 (Figure 4a), was discovered and structurally characterized using X-ray crystallography in 1982. M146791 was extracted from *Streptomyces* sp. NCIB 11649, an aerobic bacterium that had been the subject of research due to its antifungal properties. Strain NCIB 11649 was originally isolated from soil sourced in Stretford, Greater Manchester, UK. Its distinctive side group, featuring a cyclopropane terminus, had been unprecedented thus far. Cyclizidine M146791 was isolated from a liquid culture of NCIB11649 through ethyl acetate extraction at pH 7, followed by treatment with animal charcoal and recrystallization from ethyl acetate [16]. In 2015, the cyclizidine biosynthetic machinery was elucidated on the basis of this strain, based on sequencing data that resulted in a genome length of approximately 8.1 Mbp [35].

In the original publication, M146791 was depicted as (+)-ent-cyclizidine [16]. With progress in X-ray crystallography, the absolute configuration of the original sample was reassessed in 2010 and determined to be (−)-ent-cyclizidine (Figure 4b) [36]. This was achieved during attempts at the chemosynthesis of cyclizidine. One enantiomer, (+)-ent-cyclizidine, has been successfully produced in a 26-step total synthesis starting from d-serine. The overall yield was 2.7%. A significant challenge was posed by the six stereocenters (C1, C2, C3, C7, C8, and C8a). However, a stereoselective route was established, resulting in (+)-ent-cyclizidine, an enantiomer of the natural product [36]. (+)-ent-cyclizidine was also isolated from another *Streptomyces* strain, HNA39, in 2018 (see Section 3.2.) [12].

#### 3.1.2. Biosynthesis

**Figure 5:**
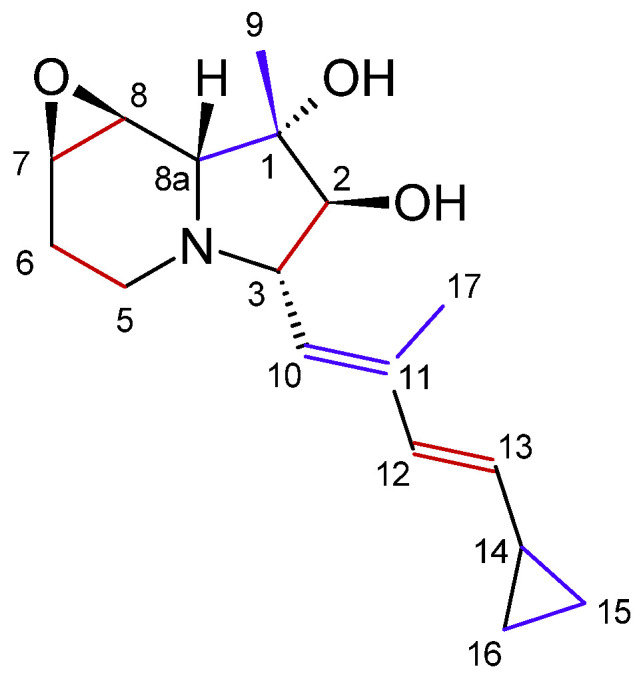
Numbering of the cyclizidine carbons and incorporation of acetate (red) and propionyl (blue) precursors in the cyclizidine structure, as proposed in [37] (modified by the author).

The first successful attempts at elucidating cyclizidine M146791 biosynthesis were performed by Leeper et al. Strain NCIB 11649 was fed with the labelled cyclizidine precursor molecules CH_3_^13^C^18^O_2_Na and CD_3_CH_2_^13^CO_2_Na. The experiments confirmed that, as per usual for polyketides, the molecule is built from acetate and propionyl units (Figure 5). The hydroxyl group at C2 is thereby derived from an acetyl moiety, while the typical cyclopropyl ring originates from a single propionate unit [37,38].

Indolizidines are synthesized by large modular enzyme complexes known as polyketide-synthases (PKSs) [35]. PKSs serve as assembly lines for numerous bioactive compounds, such as the antibiotic erythromycin [39] and the chemotherapeutic agent epothilone [40]. Three types of PKSs have been identified thus far [41]. The cyclizidine-PKS belongs to type II [35], which is characteristic for bacteria and consists of several modules, which are further subdivided into domains with specific functions. The polyketide structure is passed from module to module for processing. In each module, the polyketide is elongated and modified depending on the domains present. Typically, one module contains the obligatory ketosynthase- (KS), acyltransferase- (AT), and an acyl carrier protein (ACP) domain. The ACP transfers the growing polyketide chain to the KS of the following module. The AT elongates the chain by one carboxyacyl extender unit via esterification. Each module can include optional domains, which consecutively reduce the added extender unit: the ketoreductase (KR) reduces the added keto group to a hydroxy group, the dehydaratase-domain (DH) eliminates the hydroxy group to establish a double bond, and the double bond can be converted into a fully reduced single bond by the enoyl-reductase (ER) domain. Additionally, a methyl group can be added via the methyltransferase domain. A terminal reductase domain (TR) releases the polyketide chain from the PKS [41].

**Figure 6:**
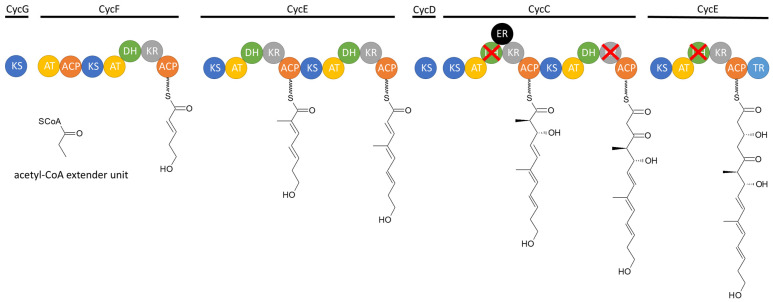
Schematic of the suggested cyclizidine PKS assembly line from strain NCIB 11649 [35] (modified by the author). KS = ketosynthase, AT = acyltransferase, ACP = acyl-carrier-protein, DH = dehydratase, KR = ketoreductase, ER = enoylreductase. The domains crossed in red are non-functional.

The cyclizidine PKS in strain NCIB 11649 has been elucidated by in vitro biochemical analysis as well as in vivo mutagenesis studies. The cluster is made up of seven modules, which are encoded in six genes, named cycB to cycG (Figure 6). Module 1 recruits the first acetyl-Coenzyme A (CoA) starter unit without further reduction of the unit. Module 2 includes a KR and DH domain, which results in the formation of a double bond in the second extender unit. This is also true for modules 3 and 4. Module 3 uses methylmalonyl-CoA as the elongation unit, which introduces an additional methyl group. Module 5 contains not only a KR and DH domain but also an ER domain. However, since the DH domain is not functional the modification of the extended unit of the polyketide stops at the hydroxy group. The KR domain in module 6 is also not functional, which results in a keto group there. Finally, in module 7, one last hydroxy group is added before the chain is released via the TR domain. The resulting chain is then modified further outside of the PKS. Additional open reading frames code for an aminotransferase, multiple oxidoreductases, a flavin reductase, and a ribonucleotide reductase, which collectively catalyze the cyclization reactions. This leads to the formation of the indolizidine core structure and the name-giving cyclopropane ring. The further biosynthetic pathway after the formation of the polyketide chain has yet to be experimentally verified [35].

#### 3.1.3. Bioactivity

After isolating the pure compound, it was confirmed that cyclizidine was not responsible for the antifungal activity of strain NCIB 11649 after all. However, cyclizidine did show non-selective immunostimulatory properties. Furthermore, the acetylated compound elicited a decrease in the beating frequency of cultured heart cells, a response reminiscent of certain P-blocking medications [16].

### 3.2. Cyclizidines from Streptomyces sp. HNA39

#### 3.2.1. Structure and Isolation

**Figure 7:**
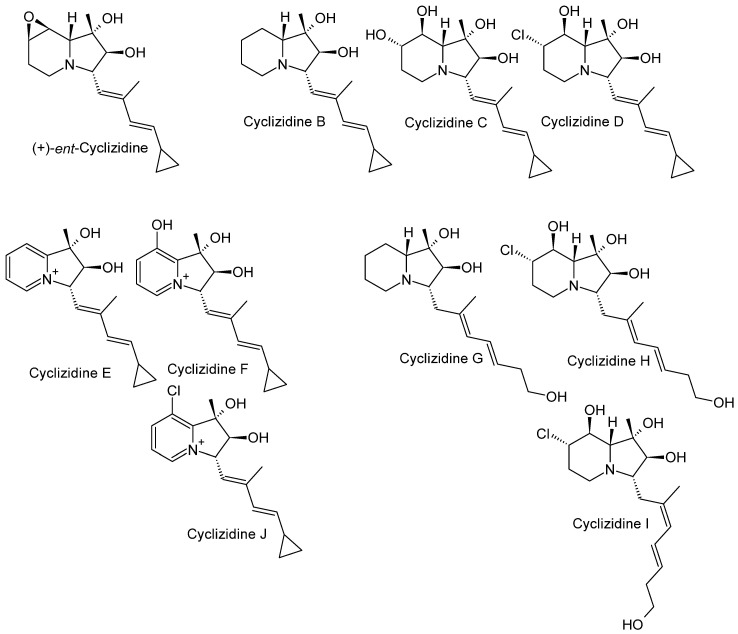
Structures of the 10 cyclizidines isolated from *Streptomyces* sp. HNA39 [12,43].

The marine *Streptomyces* sp. HNA39 originates from marine sediments of Hainan Island, China, and is part of a larger collection of 442 actinomycetical strains. As is the case with the majority of actinomycetes, this bacterium is Gram-positive, filamentous, non-motile, heterotrophic, and aerobic. It exhibits optimal growth at 28 °C, a pH of 7.0, and a low salinity of 0 to 6.5% (*w*/*v*). The initial focus on strain HNA39 was due to its capacity to produce natural products and antineoplastic properties. In addition to the discovery of new cyclizidines, bafilomycins were also identified among these natural products. The whole genome of the marine organism has recently been sequenced. The genome revealed the presence of 29 putative biosynthetic gene clusters encoding secondary metabolites. Under culture conditions, 27 of the clusters remained silent or were functionally incomplete. One cluster could be linked to bafilomycin production. Another one of these clusters is similar to the cyclizidine gene cluster of *Streptomyces* sp. NCIB11649 [42]. A total of ten cyclizidine compounds, nine of which were previously unknown, were isolated from strain HNA39. These compounds were named cyclizidine B to I (Figure 7). The previously identified compound was found to be (+)-ent-cyclizidine, which is the enantiomer of the natural cyclizidine M146791 [12]. This compound was initially isolated from NCIB 11649 [16] and has since been produced in a total synthesis [36] (see Section 3.1.1). Consequently, the structure elucidation of this compound from HNA39 was facilitated by the established NMR, HRSIMS, and specific rotation data of (+)-ent-cyclizidine [12]. It is noteworthy that the two strains NCIB 11649 and HNA39 appear to express PKSs that produce two enantiomers of the same molecule.

To isolate the compounds, strain HNA39 was fermented in liquid medium enriched with artificial seawater and the culture broth was extracted with ethyl acetate. The extract was purified by silica-gel column chromatography and the compounds were separated by HPLC. Their common structural feature, besides the indolizidine core structure, is the cyclopropane ring after which all compounds are named. Although cyclizidines G, H, and I do not show this feature, one can imagine that these two are precursors of the others, whereas G, H, and I would undergo a ring closure between C14 and C16. Cyclizidine I is the Z-isomer of cyclizidine H. Cyclizidines E, F, and J exhibit an indolizinium system that has not previously been observed in cyclizidines. The nitrogen atom at the ring bridge undergoes a transformation into a positively charged iminium cation under physiological conditions [12,43]. Cyclizidine B appears relatively unadorned, whereas cyclizidines C and D are decorated with hydroxy groups and a chlorine atom at C7 and C8, respectively. A similar observation can be made when comparing cyclizidines H and I to cyclizine D [12,43]. In (+)-ent-cyclizidine, the two adjacent groups are combined in an epoxide ring [16]. Jiang et al. do not address the question of whether these are biosynthetic by-products or artifacts of the isolation and structure elucidation procedure. Kim et al. highlight the epoxide moiety as a potential liability of the molecule during the total synthesis of an indolizidin, indolizomycin (see Section 3.5). The authors report that the molecule undergoes extensive degradation within hours at room temperature and neutral conditions [44]. It is possible that cyclizidine C is the result of such a degradation. Cyclizidines D, H, and I, however, have been chlorinated at C7 in addition to the ring opening. The use of CHCl_3_ and CDCl_3_, respectively, has been reported to cause ring opening reactions due to the formation of acidic HCl or DCl, for example, by Hamburger et al. [45]. However, no such solvents were employed during the procedure, suggesting that the chlorinated cyclizidines may well be natural products [12].

#### 3.2.2. Bioactivity

Cyclizidines B to I, as well as (+)-ent-cylizidine, were tested for their bioactivity against the cancer cell lines HCT-116 (colon cancer) and PC-3 (prostate cancer) [12] in a colorimetric cytotoxicity assay with sulforhodamine B (SRB) [46]. Cyclizidine J was tested only against PC-3 [43]. The compounds showed low to moderate activities, with only cyclizidine C showing high cytotoxicity with IC_50_ values of 0.52 ± 0.003 µM against PC-3 and 8.3 ± 0.1 µM against HCT116, while the positive control staurosporine reached values of 0.017 ± 0.004 and 0.055 ± 0.001, respectively. In addition, in a ROCK2 protein kinase inhibitory assay not only cyclizidine C but also F, H, and I achieved moderate IC_50_ values [12,43].

A comparison of the bioactivities of the two enantionmers of cyclizidine produced by the two strains NCIB 11649 and HNA39 would be a valuable addition to the existing research. Thus far an array of disparate properties have been assessed. However, a direct comparison of cytotoxicity and immunostimulatory properties against identical cell lines would be highly beneficial. Furthermore, elucidating the biosynthetic machinery of (+)-ent-cyclizidine from HNA39 would be beneficial for evaluating the structural differences in the PKSs that result in distinct enantiomers.

### 3.3. Cyclizidines from Saccharopolyspora sp. RL78

#### 3.3.1. Structure and Isolation

**Figure 8:**
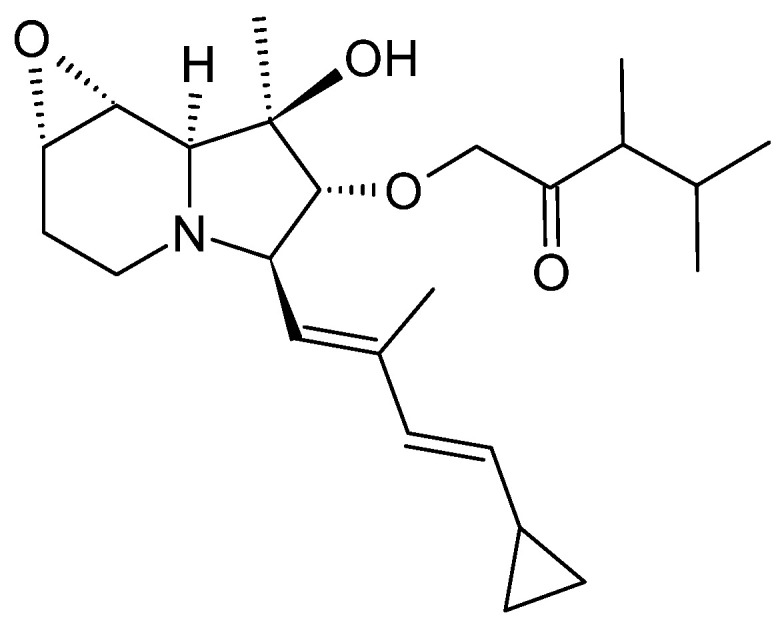
Structure of the cyclizidine JBIR-102 from *Saccharopolyspora* sp. RL78 [13].

Izumikawa et al. searched for new therapeutics against malignant pleural mesothelioma (MPM). To this end, they screened the butanol culture extracts of 347 newly isolated strains from terrestrial and marine habitats by high-performance liquid chromatography and mass spectrometry for peaks not accounted for in the house internal database. The unknown compounds were isolated and their structures elucidated. Among the newly identified compounds was a new cyclizidine derivative, named JBIR-102 (Figure 8), produced by *Saccharopolyspora* sp. RL78. This species was isolated from a mangrove soil sample from Ishigaki Island, Japan [47]. *Saccharopolyspora* is a minor genus among the actinomycetes, comprising just over 30 species. Nevertheless, they are regarded as prolific producers of secondary metabolites. It is noteworthy that they appear to have a preference for extreme environments: 16 *Saccharopolyspora* species from extreme marine habitats are classified as halophilic or at least halotolerant, while those from terrestrial environments are often described as thermophilic, alkaliphilic, and halophilic [48].

For compound isolation, strain RL78 was cultivated in a sea salt-enriched liquid medium. After extraction of the cell pellet with acetone and ethyl acetate and separation by HPLC, an isobutyl ester derivative of (−)-ent-cyclizidine was isolated [13]. The stereochemistry is, thus, identical to the revised version of cyclizidine M146791 (Figure 4) [13,36]. To date, the biosynthetic machinery responsible for the production of JBIR-102 remains unidentified, and no bioinformatical studies have been published. However, the striking similarities between JBIR-102 and cyclizidine M146791 suggest a nearly identical assembly line.

#### 3.3.2. Bioactivity

The activity of JBIR-102 against malignant pleural mesothelioma and cervical cancer cells was tested in a colorimetric assay. JBIR-102 showed an IC_50_ value of 39 µM against MPM ACC-MESO-1 cells and 29 µM against HeLa cells. In comparison, cyclizidine shows slightly better activity with IC_50_ values of 32 µM and 16 µM, respectively [13].

### 3.4. Iminimycins from Streptomyces griseus

#### 3.4.1. Structure and Isolation

**Figure 9:**
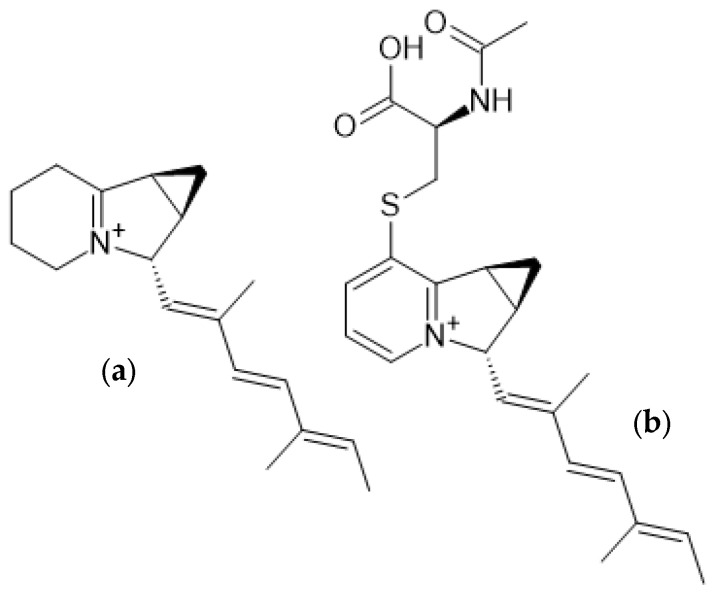
Structures of (**a**) iminimycin A and (**b**) iminimycin B from *S. griseus* OS-3601 [15,34].

Studies on the abilities of *S. griseus* strains to produce secondary metabolites have been conducted since the early days of antibiotic drug discovery [49]. With regard to this matter, *S. griseus* has long been known to be a producer of streptomycin [24]. Recently, two novel nonribosomal peptides, grisgenomycin A and B, were isolated from *S. griseus* species NBRC 13350 and ATCC 12475 [50]. Furthermore, in 2016, two previously unknown indolizidines were isolated from *S. griseus* OS-3601, which had been stored freeze-dried for 40 years before being reactivated for this study. Isolation from culture broth was performed by silica-gel column chromatography followed by HPLC. The iminimycins were eluted with methanol in water. The core of the isolated structures consists of a 6-5-3 tricyclic system. Both molecules, named iminimycin A and B, carry a rare iminium cation and a cyclopropyl moiety fused to the indolizidine structure (Figure 9) [15,34]. The iminium cation can also be found in cyclizidines E, F [12], and J [43] (Figure 7). Unlike many cyclizidines (Figure 4, Figure 7, and Figure 8), iminimycins A and B do not carry a cyclopropyl moiety at the olephinic tail, nor are the molecules hydroxylated or chlorinated. The cyclohexane ring in iminimycin B is aromatized and an N-acetyl-cysteine moiety is attached to the aromatized indolizidine ring. The stereochemistry at C3 is consistent with that of (+)-ent-cyclizidine as produced by HNA39 (Figure 7) [15,34].

#### 3.4.2. Biosynthesis

**Figure 10:**
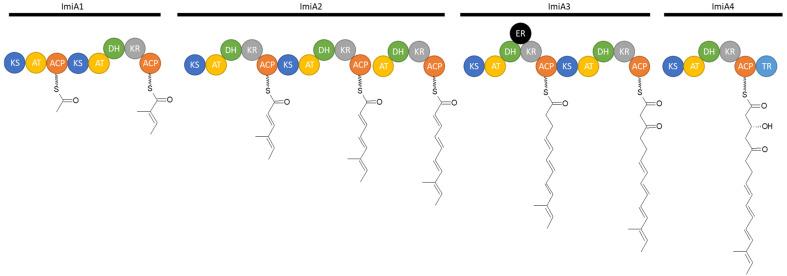
Schematic of the suggested iminimycin PKS assembly line from *S. griseus* OS-3601 ([51], modified by the author). KS = ketosynthase, AT = acyltransferase, ACP = acyl-carrier-protein, DH = dehydratase, KR = ketoreductase, ER = enoylreductase.

In addition to the isolation of these two compounds, the biosynthetic pathway of iminimycins was elucidated by bioinformatic analysis and subsequent inactivation of several genes within the biosynthetic gene cluster. Bioinformatic analysis revealed 22 genes, named *imiA* to *imiT*. Four genes, *imiA1* to *4*, encode a type I PKS (Figure 10) that synthesizes the polyketide chain on which the iminimycin structure is based. Eight modules forge the iminimycin chain. The acyltransferase domain of the first module was predicted to select (2*S*) methylmalonyl-CoA as the starter unit [51].

**Figure 11:**
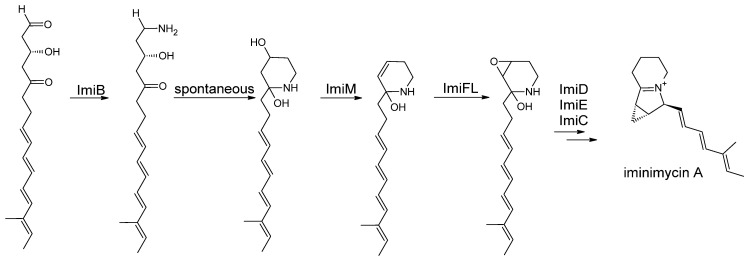
Post-PKS modifications of iminimycins in *S. griseus* OS-3601 ([51], modified by the author).

Eight modules in the gene cluster form a 16-membered polyketide chain: a tetraene is followed by modules with a fully oxidized keto moiety and a hydroxy group. After release, the group terminates in an aldehyde moiety. This aldehyde moiety is aminated by ImiB (Figure 11). The aminated polyketide chain undergoes a spontaneous cyclization reaction forming the six-membered ring of the indolizidine structure. Elimination of the hydroxy group at C4 results in a C4C5 double bond, which is oxidized to form the epoxide ring. The subsequent reactions are expected to be catalyzed by ImiM and ImiFL, respectively. The following ring formation reaction, which completes the indolizidine core structure and results in iminimycin A, is catalyzed by ImiD, C, and E [51].

#### 3.4.3. Bioactivity

Iminimycin A showed little antibiotic activity against the bacteria *Bacillus subtilis*, *Kocuria rhizophila*, and *Xanthomonas campestris pv. oryzae*. Cytotoxicity against HeLa S3 and Jurkat cells was also determined, with IC_50_ values of 43 µM and 36 µM, respectively [15].

### 3.5. Indolizomycin from Streptomyces sp. SK2-52

#### 3.5.1. Structure and Isolation

**Figure 12:**
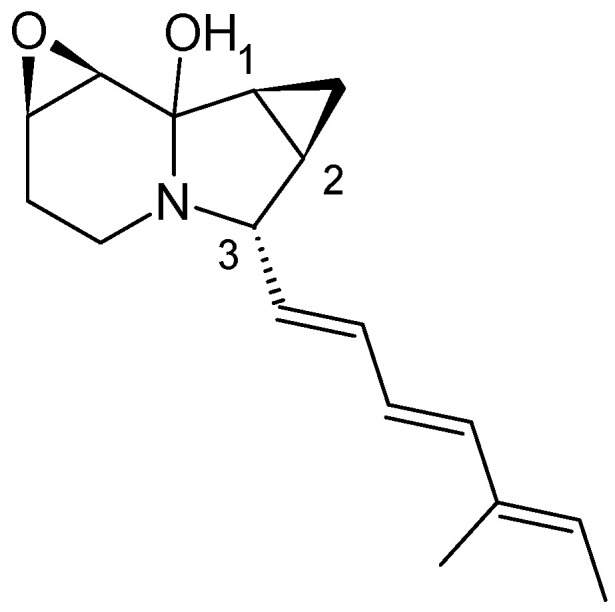
Structure of indolizomycin from strain SK2-52 [14].

Only two years after the discovery of the first indolizidine, cyclizidine M146791, from an actinomycete species [16], a second indolizidine, named indolizomycin, was discovered. Like cyclizidine, indolizomycin contains a cyclopropyl ring, not at the end of the olephinic tail but as a third ring added to the cyclopentane ring of the core indolizidine-structure, similar to the iminimycins (Figure 12). The supposedly open cyclopropane unit at the end of the olephinic tail, as well as the stereochemistry at C1, C2, and C3, also exhibits a resemblance to those of the iminimycins [14].

This metabolite was not produced by a wild-type strain, but by a strain produced by interspecies fusion treatment: protoplasts of *S. griseus* SS-1198, which was unable to produce any bioactive compounds (including iminmycins), were fused with *S. tenjimariensis* SS-939 [14]. *S. tenjimariensis* is a known istamycin producer [52]. As a consequence of the interspecies fusion, the transformed strain *S. griseus* SK2-52 inherited resistances against streptomycin and kanamycin from *S. griseus* and *S. tenjimariensis*, respectively. During the growth phase, strain SK2-52 exhibited morphological characteristics similar to those of *S. griseus*. Most importantly, *S. griseus* SK2-52 was capable of producing indolizomycin, a capability that was not exhibited by either of the parental strains [14]. Nevertheless, indolizidine compounds have been isolated from a strain of *S. griseus* before [15], which suggests that perhaps the protoplast fusion has activated a silent gene cluster in *S. griseus* SK2-52 [44]. The indolizomycin was extracted from the culture broth using an Amberlite resin column and eluted with 30% aqueous acetone. Subsequent treatment of the crude lyophilizate with methanol and preparative TLC purification followed by ethyl acetate extraction yielded a pure compound [14].

Total chemosynthesis of indolizomycin has been conducted. A significant challenge was posed by the instability of indolizomycin, the cause of which remains unclear. The authors proposed that introduction of the triene and the carbinol amine could, at a late stage, reduce the problem. This approach resulted in the synthesis of racemic indolizomycin with confirmed stereochemistry with a yield of 29% [44,53].

#### 3.5.2. Bioactivity

Indolizomycin exhibits weak antibiotic activity against several bacterial and fungal strains, such as Staphylococci, Bacilli, Escherichia coli, and Candida albicans. In addition, the LC_50_ values against mice were determined to be 12.5 to 25 mg/kg. Cytotoxicity has not been tested [14].

## 4. Actinomycetes with a Silent Cyclizidine Cluster

Only a limited number of bacterial indolizidine producers have been identified thus far. However, it is probable that additional ones exist, harboring the potential to produce novel and bioactive compounds. Bioinformatic tools provide the means to screen huge amounts of genomic data, which simplifies the search for novel indolizidine producing strains. To give an example: In *Streptomyces* sp. NCIB 11649 the gene cluster encoding the corresponding cyclizidine was elucidated (Figure 6). This biosynthetic gene cluster can be used to identify other potential cyclizidine producers. The cluster has already been identified with almost 100% identity in *Actinosynnema mirum* DSM 43827 [35]. *A. mirum* was first described and designated a type strain for its genus in 1978. The strain was isolated from a grass blade at the Raritan River, New Jersey and named after the erect and often fused hyphae (synnemata), which are a common phenomenon in actinomycetes. The publication already included a description of the strain’s antibiotic properties against Gram-positive bacteria and some eukaryotes [54]. Subsequently, it was discovered that *A. mirum* is a producer of nocarcidin β-lactam antibiotics [55]. It is noteworthy that spores of *A. mirum* form flagella and are, thus, motile, in contrast to the majority of actinomycete spores, which are immotile [54]. The genome of the *A. mirum* type strain 101^T^ was fully sequenced in 2009 [56], thereby enabling bioinformatic analysis and the identification of the cyclizidine gene cluster. The PKS of this cluster lacks the ER domain in domain 5, as well as the KR and DH domains in module 6. Furthermore, two additional putative KS domains appear to be fused to the N-terminus of what corresponds to CycF and CycC in strain NCIB 11649. However, the cyclizidine cluster in *A. mirum* is not expressed under laboratory conditions. Thus, what kind of cyclizidine this altered cluster encodes and what bioactivities it might have remain to be explored [35]. Another potential candidate as a cyclizidine producer is *Streptomyces venezuelae* ATCC 150068. *S. venezuelae* is a strain that played a pivotal role in the early days of antibiotic drug discovery. It was first described as a producer of chloromycetin in 1948. The strain was named after its collection source, which was a mulched field near Caracas in Venezuela [57]. A cluster comparison with the cluster-finding tool antiSMASH [58] revealed a cyclizidine cluster with 82% similarity (unpublished data).

## 5. Conclusions

Collectively, the presented findings demonstrate that indolizidine derivatives have the potential to become anticancer drug candidates. However, there is currently a significant gap in our knowledge of these structures, particularly with regard to their pathway after release from polyketide synthases, the formation of the cyclopropane moiety in cyclizidines, and, most importantly, their mode of action.

Furthermore, it is plausible that additional producers of these compounds, akin to *A. mirum* or *S. venezuelae*, exist. Activation of silent gene clusters could reveal new indolizidine derivatives with novel bioacitivities. The exploration of novel derivatives is warranted, as bacteria have been demonstrated to generate a diverse array of bioactive and potentially beneficial molecules. While the search for bacterial indolizidines, with potential applications as anticancer agents, holds promise, the outcome remains uncertain, and success may ultimately hinge on chance as it always does with natural products from microorganisms.

## Data Availability

Not applicable.
